# A song of heads and tails: myosin II conformational regulation and filament dynamics shape force generation in non-muscle cells

**DOI:** 10.1007/s12551-026-01414-1

**Published:** 2026-03-10

**Authors:** Rafael Pérez-Díaz, Marina Garrido-Casado, Hugo Ramos-Solano, Clara Llorente-González, Vanessa C. Talayero, Miguel Vicente-Manzanares

**Affiliations:** Molecular Mechanisms Program, Centro de Investigación del Cáncer, Consejo Superior de Investigaciones Científicas (CSIC), Universidad de Salamanca, Salamanca, Spain

**Keywords:** Myosin, Actin, Cross-bridge, Muscle, Smooth muscle, Non-muscle, Stress fiber, HCM, DCM, MYH9-RD, Mavacamten

## Abstract

Non-muscle cells generate force without forming sarcomeres, building instead highly dynamic, contractile filaments that assemble, remodel, and disassemble in response to mechanical and biochemical signals. This review focuses on the conformational regulation and filament dynamics of myosin II paralogs as they define diverse types of cytoplasmic structures that produce mechanical forces. Whereas muscle myosin II stably resides in sarcomeres and conserve energy by adopting a super-relaxed state in which myosin II heads interact with each other and the core of the thick filament, smooth muscle and non-muscle myosin II shift between a soluble, folded, auto-inhibited 10S species and filaments, where they adopt an extended, assembly-competent 6S form. Phosphorylation of smooth muscle and non-muscle regulatory light chain triggers the conformational transition from 10S to 6S, leading to filament formation and contractile output. Other phosphorylations in the regulatory light and heavy chains also control filament assembly and dynamics through different molecular mechanisms. Biochemical and mechanical inputs fine-tune filament size, lifetime, and duty ratio, shaping contractile output across diverse cellular contexts. Upstream regulators, including biochemical and mechanical inputs, converge on several pathways, e.g., Ca^2+^/MLCK and RhoA/ROCK, organizing myosin II activity in space and time and enabling the emergence of stress fibers, junctional belts, cortical networks, and contractile rings that support adhesion, migration, cytokinesis, and tissue-level mechanics.

## Introduction

Contractile forces are universal drivers in cell biology, propelling macroscopic movements such as voluntary muscle action, cardiac contractions, sphincter and bowel motility, or newborn egress from the womb. Similar forces also power cellular-level events, including cell–cell communication, migration, mitosis, and organelle positioning (Fletcher and Mullins, 2010). Most contractile forces are generated by proteins of the myosin superfamily (Berg et al., 2001), specifically class II myosins (Rassier and Mansson, 2025). Class II myosins are highly conserved through eukaryotic evolution (Odronitz and Kollmar, 2007). They are often categorized according to their tissue-specific expression in metazoans into striated muscle (skeletal and cardiac), smooth muscle, and non-muscle types (Coluccio, 2020).

Structurally, the functional myosin II monomer is a hexamer consisting of two heavy chains that confer isoform-specific identity, two essential light chains that stabilize the neck region, and two regulatory light chains (Chinthalapudi and Heissler, 2024; Rassier and Mansson, 2025; Vicente-Manzanares et al., 2009). The heavy chain is a long (~ 2000 amino acids) polypeptide composed of distinct domains: a globular head domain containing the ATPase motor and actin-binding sites; a neck or lever arm region that binds the light chains and amplifies conformational changes; and a long coiled-coil tail domain that enables dimerization and filament assembly. Some myosin II heavy chain isoforms also contain a non-helical, disordered tailpiece that modulates filament formation and dynamics (Rassier and Mansson, 2025; Vicente-Manzanares et al., 2009).

Various genes encode muscle myosin 2 (MM2) heavy chains and light chains. Multiple genes encode myosin 2 heavy chains selectively expressed in striated muscle (*MYH1*, *MYH2*, *MYH4*), cardiac muscle (*MYH6*, *MYH7*), or in specific muscle groups, such as the extraocular muscles (*MYH15*). Others are expressed during development (*MYH3*) and regeneration (*MYH8*). Smooth muscle myosin II heavy chain (SM-MHC2) is encoded by a single primary gene (*MYH11*), and non-muscle myosin II heavy chains (nmMHC2-A/B/C) are encoded by three genes (*MYH9*, *MYH10*, *MYH14*) (Chinthalapudi and Heissler, 2024; Heissler and Manstein, 2013; Weiss et al., 1999). Several genes encode essential (structural) light chains (ELCs) and regulatory light chains (RLCs) (Lowey and Trybus, 2010), though not all combinations form functional monomers (Sitbon et al., 2020).*MYL12A/B* genes encode the non-muscle RLC isoforms and are expressed in most tissues, whereas *MYL9 *encodes the smooth muscle isoform (Heissler and Sellers, 2014). It is worth noting that MYL9’s protein product is also expressed in cells other than smooth muscle, e.g., platelets (Gilles et al., 2009; Jalagadugula et al., 2010), normal fibroblasts (Esnault et al., 2014), cancer-associated fibroblasts (Calvo et al., 2013; Foster et al., 2017), melanoma cells (Luo et al., 2014; Medjkane et al., 2009), and other cell types (Park et al., 2011). *MYL6*, the gene encoding the non-muscle/smooth muscle essential light chain, is expressed ubiquitously (Olivieri et al., 2021). Each MM2/SM2 (smooth muscle myosin 2)/NM2 (non-muscle myosin 2) monomer has different specific properties that are attuned to the function of the cells that express them. Nomenclature-wise, monomer identity is defined by the heavy chain isoform, and monomers bearing different heavy chains are termed paralogs. The NM2 paralogs are a representative example: nmMHC2-A is encoded by gene *MYH9*; *MYH10* encodes nmMHC2-B; and *MYH14* encodes nmMHC2-C. The combination of protein products from genes *MYH9* + *MYL12A/B* + *MYL6* is the NM2-A paralog; *MYH10* + *MYL12A/B* + *MYL6* is the NM2-B paralog; and *MYH14* + *MYL12A/B* + *MYL6* is the NM2-C paralog.

The genetic organization of key mammalian myosin II paralogs and heavy chain isoforms and their functional ELC/RLC combinations are summarized in Table 1. Splicing variants are not included here (except *MYL12A/B* and *MYL6/6B*), but there is evidence of crucial physiological roles for spliced variants of, for example, nmMHC2-A, B, and C (Das et al., 2023; Jana et al., 2009; Kim et al., 2008).

**Table 1:** Genetic and molecular organization of myosin 2 paralogs

Myosin paralog	HC	HC gene	RLC	RLC gene	ELC	ELC gene	Refs
NM2-A	nmMHC2-A	*MYH9*	nmRLC, MRLC2, MRLC3 smRLC, MRLC1, RLC2	*MYL12A/B* *MYL9*	ELC/MLC1SA	*MYL6/6B*	(Chinthalapudi and Heissler, 2024; Conti et al., 2007)
NM2-B	nmMHC2-B	*MYH10*	nmRLC, MRLC2, MRLC3 smRLC, MRLC1, RLC2	*MYL12A/B* *MYL9*	ELC/MLC1SA	*MYL6/6B*	(Chinthalapudi and Heissler, 2024; Conti et al., 2007)
NM2-C	nmMHC2-C	*MYH14*	nmRLC, MRLC2, MRLC3 smRLC, MRLC1, RLC2	*MYL12A/B* *MYL9*	ELC/MLC1SA	*MYL6/6B*	(Chinthalapudi and Heissler, 2024; Conti et al., 2007)
Smooth muscle (SM2)	smMHC2	*MYH11*	smRLC, MRLC1, RLC2	*MYL9*	ELC	*MYL6/6B*	(Cremo and Hartshorne, 2007)
Cardiac muscle α-myosin atrial	α-cardiac MHC2	*MYH6*	MYL7 MYLC2a	*MYL7*	AMLC, ALC1	*MYL4*	(Rassier and Mansson, 2025; Reggiani and Bottinelli, 2007)
Cardiac muscle β-myosin ventricular	β-cardiac MHC2	*MYH7*	MLC-2 s/v, MLC-2v	*MYL2*	MLC1V, VLC1	*MYL3*	(Rassier and Mansson, 2025; Reggiani and Bottinelli, 2007)
Skeletal muscle (slow, type I)	MyHC-I (slow twitch, high efficiency) MYH14 (slow tonic)	*MYH7* *MYH14*	MLC-2 s/v, MLC-2v	*MYL2*	MLC1V, VLC1 MLC1SA	*MYL3* *MYL6B*	(Rassier and Mansson, 2025; Reggiani and Bottinelli, 2007)
Skeletal muscle (fast, type IIX fibers)	mMHC2X, MyHCII-d/x	*MYH1*	MYLPF, MLC2	*MYL11*	MLC1	*MYL1*	(Rassier and Mansson, 2025; Reggiani and Bottinelli, 2007)
Skeletal muscle (fast, type IIa fibers)	mMHC2A MyHC-IIa	*MYH2*	MYLPF, MLC2	*MYL11*	MLC1	*MYL1*	(Rassier and Mansson, 2025; Reggiani and Bottinelli, 2007)
Skeletal muscle (fast, type IIb fibers)	mMHC2B MyHC-IIb	*MYH4*	MYLPF, MLC2	*MYL11*	MLC1	*MYL1*	(Rassier and Mansson, 2025; Reggiani and Bottinelli, 2007)
Extraocular, super-fast	Extraocular	*MYH13* *MYH15*	MYLPF, MLC2	*MYL11*	MLC1	*MYL1*	(Lee et al., 2019)
Embryonic	Emb	*MYH3* *MYH14*	MLC-2 s/v, MLC-2v MYLPF, MLC2	*MYL2* *MYL11*	AMLC, ALC1	*MYL4*	(Zhao et al., 2022)
Perinatal	Peri	*MYH8*	MLC-2 s/v, MLC-2v MYLPF, MLC2	*MYL2* *MYL11*	AMLC, ALC1	*MYL4*	(Agarwal et al., 2020)

The references correspond to general reviews focused on the specific paralog(s) indicated

This review highlights key differences in contractile force generation mechanisms among MM2, SM2, and NM2. It illustrates NM2-specific regulatory modes that explain its role in cellular plasticity, linking the biophysics of force generation to relevant biochemical and molecular mechanisms in molecular and cellular contexts.

## Molecular differences in force generation between striated muscle, smooth muscle, and non-muscle cells

Striated muscle myosin II (MM2) powers contraction and large-scale force production, including heartbeat dynamics and voluntary muscle flexing and extension. Detailed mechanisms of MM2-driven force have been reviewed elsewhere (Rassier, 2017; Rassier and Mansson, 2025). In skeletal fibers, electrical impulses propagate from the cell membrane into T-tubules, exciting dihydropyridine receptors (DHPR). These physically link to ryanodine receptors (RyR) embedded in the sarcoplasmic reticulum (SR), prompting swift cytosolic Ca^2^⁺ discharge within milliseconds. Cardiac cells differ: DHPR opening enables external Ca^2^⁺ influx, which then stimulates RyR via calcium-triggered release (CICR)—yielding transients roughly tenfold weaker than skeletal responses (Brunello and Fusi, 2024; Cho et al., 2017). Elevated Ca^2^⁺ engages troponin C along thin filaments, reshaping the troponin-tropomyosin complex to unmask actin’s myosin binding sites (Gordon et al., 2000; Rassier and Mansson, 2025). MM2 heads then attach to actin and ATP hydrolysis fuels the power stroke and repetitive cross-bridge motion (Geeves and Holmes, 1999; Rassier and Mansson, 2025; Spudich, 2001). By targeting multiple troponin sites at once, Ca^2^⁺ sparks synchronized MM2 engagement across the sarcomere array, yielding potent, unified forces from a lone nerve signal (Brunello and Fusi, 2024; Rassier and Mansson, 2025).

Smooth muscle is activated predominantly by autonomic nerve fibers that form neuromuscular junctions with the smooth muscle cell layer. Neurotransmitters such as acetylcholine and norepinephrine are released from varicosities along these fibers and diffuse across the cell junctions, binding G protein–coupled receptors on smooth muscle cells (Brozovich et al., 2016). Ligand binding triggers membrane depolarization and/or IP₃ production, opening voltage-gated and receptor-operated Ca^2^⁺ channels that mediate Ca^2^⁺ release from the sarcoplasmic reticulum, thereby elevating cytosolic Ca^2^⁺ (Hill-Eubanks et al., 2011); Ca^2^⁺ then binds calmodulin to form an active Ca^2^⁺–calmodulin complex that stimulates myosin light chain kinase, leading to phosphorylation of the regulatory light chain of SM2 (Somlyo and Somlyo, 2003). This sparks the ATPase activity of SM2 and engages actin–myosin cross-bridge cycling (Rassier and Mansson, 2025; Spudich, 2001). The RhoA/ROCK pathway sustains tonic activation of SM2 by inhibiting MYPT1, which maintains overall RLC phosphorylation elevated and decreases SM2 filament turnover (Randar et al., 2025). Calcium and the RhoA pathway translate neural inputs into graded, often long-lasting contractions that regulate vascular resistance, airway caliber, and visceral motility.

NM2 is typically activated not by direct synaptic input but by intracellular signaling pathways downstream of growth factors, adhesion receptors, and mechanical cues (Garrido-Casado et al., 2021). Ligand binding to receptor tyrosine kinases, G protein–coupled receptors, or integrins engages specific signaling cascades that locally elevate cytosolic Ca^2^⁺ and activate RhoA (Soriano et al., 2021). RhoA can also be activated by cellular confinement and mechanical forces in a PLA_2_-AA-dependent manner (Venturini et al., 2020). Similar to smooth muscle, Ca^2^⁺/calmodulin stimulates myosin light chain kinase, while RhoA, via ROCK, inhibits MYPT1 and can phosphorylate RLC directly. Intracellular Ca^2+^ levels are reciprocally connected to mechanical signals through the activation of mechanosensitive Ca^2+^channels such as Piezo1 (Coste et al., 2010; Yao et al., 2022). Other kinases, e.g., ZIPK, CITK, and MRCK, can phosphorylate RLC in a context-specific manner (reviewed in (Garrido-Casado et al., 2021)). These pathways induce the dynamic assembly of NM2 filaments that generate mechanical force by actin–myosin cross-bridge cycling. In specific scenarios, NM2 can form stable cortical networks, stress fibers, and contractile rings (Fernandez-Gonzalez et al., 2009; Naumanen et al., 2008; Smutny et al., 2010). The high spatial and temporal plasticity of these signaling modules patterns NM2 activation and assembly across the cell to drive processes such as edge protrusion–retraction cycles, adhesion maturation, cytokinetic furrow ingression, and tissue morphogenesis.

### Molecular features of MM2, SM2, and NM2

**Fig. 1:**
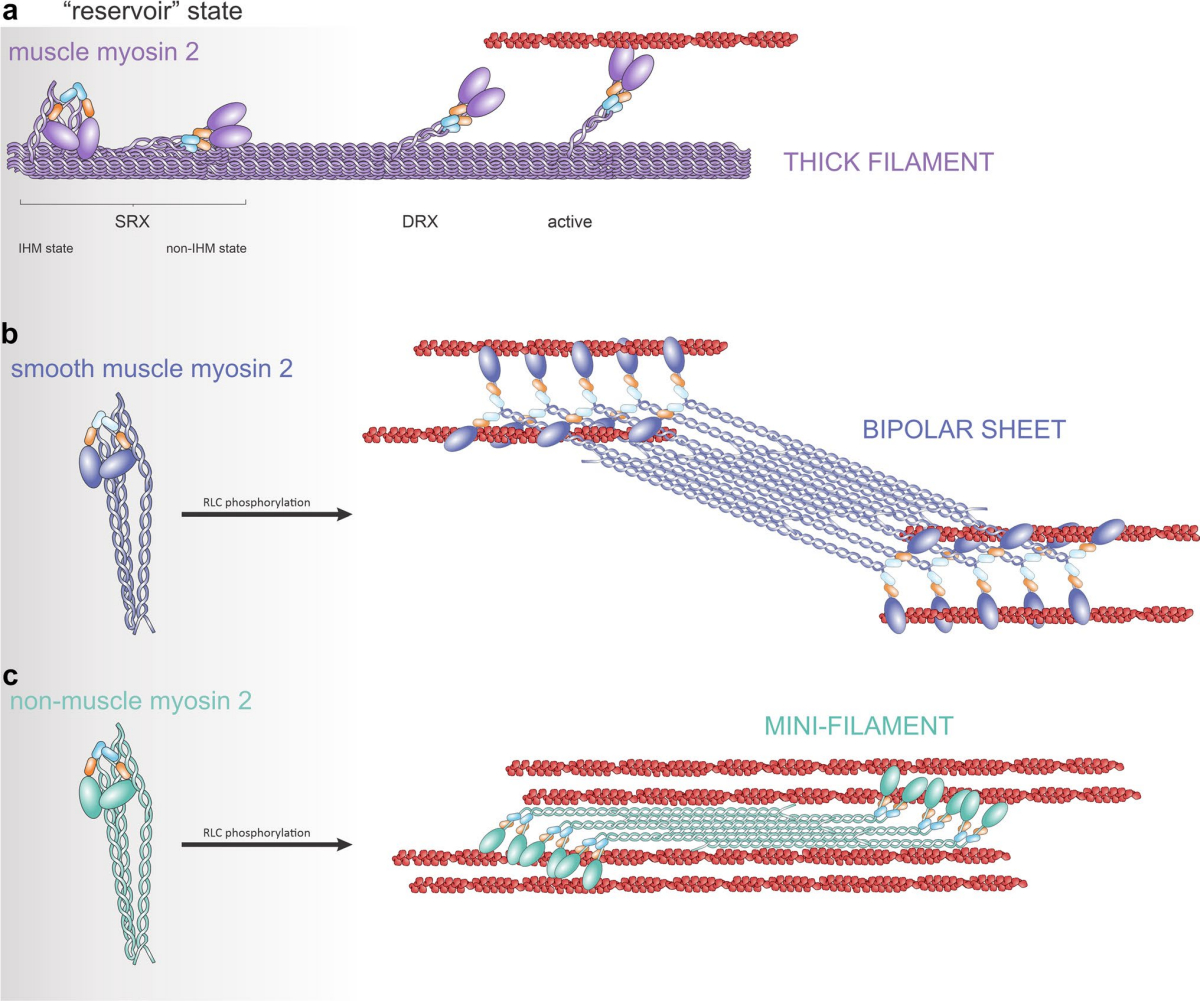
Types of myosin 2 reservoirs and assemblies. **a** In muscle cells, MM2 adopts the super-relaxed state (SRX), in which the head domains may be interacting (left, IHM state), or not, with each other and the stem of the coiled coil (IHM). ATP consumption in this state is minimal. Upon activation, the SRX state evolves into a disordered, relaxed state, where the head domains are available to interact with the thin (actin filament). Basal ATP expenditure is higher than in SRX. Finally, heads engage with F-actin in the active state, where ATP expenditure is maximal. **b** SM2 and **c** NM2 appear in a folded, cytoplasmic conformation that does not enable growth into large filaments. Upon conformational extension (See Fig. 2), SM2 forms stable bipolar sheets (**b**), and NM2 forms bipolar filaments (**c**)

In striated muscle, myosin II (MM2) adopts three primary states: (i) Active, defined by rapid ATP hydrolysis (≈5 to 10 s^−1^), yielding a high-affinity actin state (Johnson et al., 2019) (Fig.1A). Phosphate release powers the conformational shift that slides actin filaments toward the sarcomere midpoint, driving shortening (Debold et al., 2025). (ii) Disordered relaxed (DRX): ATP-bound heads detach from actin yet hover near thin filaments, exhibiting modest basal ATPase activity while primed for rebinding (Cail et al., 2025). (iii) Super-relaxed (SRX): Heads bend together forming an interacting-heads motif (IHM), with both heads folded back against the coiled-coil tail base (Mohran et al., 2024). This is an energy-saving conformation that minimizes ATP use (Lee et al., 2018). One myosin head, designated the blocked head (BH), is sterically trapped between the proximal coiled-coil tail segment and the other head, the free head (FH), preventing its interaction with actin (Grinzato et al., 2023).

SM2 and NM2 do not appear in the super-relaxed (SRX) state seen in striated MM2 because they do not form sarcomeres. Instead, these isoforms minimize ATP consumption by folding into a compact 10S conformation, where the two heads fold back intramolecularly in a manner reminiscent of the SRX interacting-heads motif (Craig et al., 1983; Heissler et al., 2021; Ikebe, 2008; Scarff et al., 2020; Yang et al., 2020) (Fig.1B–C). This folded 10S form serves a similar role to the SRX state in terms of energy conservation. However, it offers an additional advantage due to its solubility in the cytoplasm, constituting a readily accessible reserve that cells can rapidly mobilize in response to extracellular cues. Upon phosphorylation of the RLC, these monomers unfold and assemble into dynamic filaments, enabling the cell to quickly enhance its contractile machinery and adapt to changing environmental demands.

Activation of SM2 and NM2 conformational changes occurs primarily through phosphorylation of their regulatory light chains (RLC) at serine 19. First characterized over four decades ago (Craig et al., 1983), this modification introduces negative charge repulsion that disrupts interactions between the blocked head (BH), free head (FH), and coiled-coil tail (Heissler et al., 2021). The resulting electrostatic destabilization promotes head–tail dissociation, transitioning the compact 10S monomer to a 6S extended form that can form filaments (assembly-competent).

### Molecular regulation of MM2, SM2, and NM2 activity

Although multiple kinases can target Ser19 on RLC (Garrido-Casado et al., 2021), myosin light chain kinase (MLCK) serves as its primary physiological regulator (Somlyo and Somlyo, 2003). Four distinct genes (*MYLK1-4*) encode MLCK isoforms with specialized expression patterns.

*MYLK1 *produces both smooth muscle MLCK (~ 130 kDa short isoform) and non-muscle MLCK (~ 220 kDa long isoform) through alternative promoters and splicing variants (Birukov et al., 1998). *MYLK2* and *MYLK3 *generate skeletal and cardiac MLCKs, respectively, each optimized for striated muscle contractility (Stull et al., 2011). *MYLK4*, the least studied, appears to contribute to non-canonical myosin II-related signaling (Sakakibara et al., 2021).

**Fig. 2:**
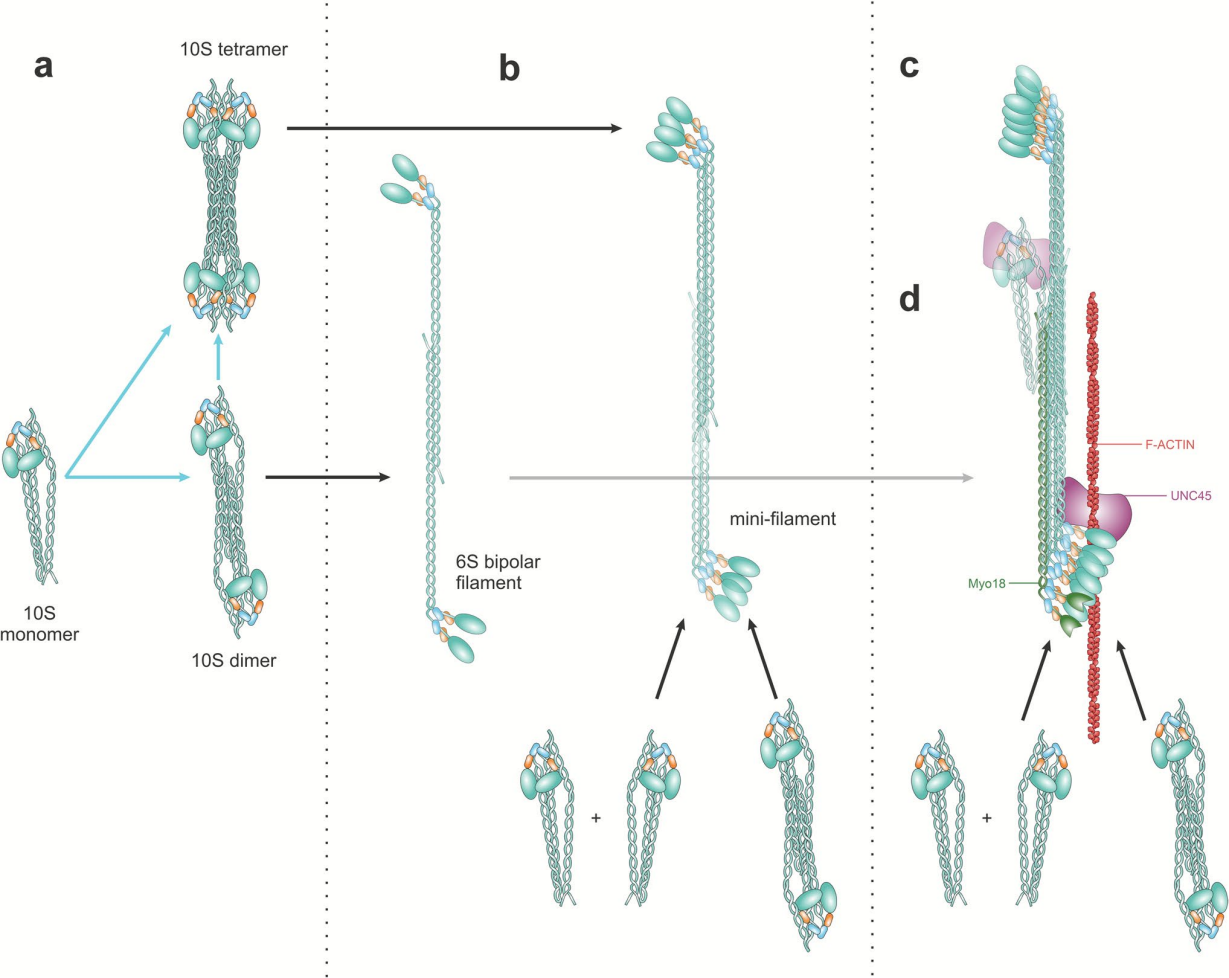
A model of SM2/NM2 filament formation. According to Korn and co-workers (Liu et al., 2017), SM2/NM2 10S monomers may form folded dimers or tetramers in the absence of RLC phosphorylation (**a**, blue arrows). Upon RLC phosphorylation (black arrows), dimers or tetramers extend into bipolar filaments with 2 or 4 heads for half-filament (**b**). These can grow by incorporation of individual 10S monomers or dimers. **c** Larger order filaments are maintained in homeostasis through incorporation of 10S monomers/dimers assisted by non-contractile Myo18 (green), directed by F-actin filaments (red) and/or assisted by UNC45a chaperone (mauve). **d** Monomers can also be exchanged through the establishment of electrostatic interactions between the exposed coiled-coils of the filament and the folded coiled-coil of monomers, also assisted by UNC45a

Single non-muscle or smooth muscle myosin II (NM2/SM2) molecules in the extended 6S state are not stable in the cytoplasm and tend either to refold into the compact 10S conformation or to assemble into higher-order structures (Ikebe, 2008). Elegant work from the Korn laboratory indicates that folded NM2/SM2 monomers first associate into antiparallel, still folded, dimers and tetramers (Liu et al., 2017) (Fig.2a). Upon Ser19 RLC phosphorylation, folded oligomers “flatten” into extended bipolar filaments (Fig. 2a–b). In these assemblies, the two heads from one myosin molecule project from one filament end, the paired heads from the antiparallel partner emerge from the opposite end, and the central shaft consists of the associated coiled-coil tails. Filaments can subsequently increase in size through two main mechanisms: local expansion of existing bipolar filaments, where additional myosin II molecules extend individual filaments along their long axis, and actin-guided concatenation, in which separate bipolar filaments are brought together and fuse into larger stacks (Fenix et al., 2016) (Fig.2c). Non-contractile myosin-18B (*MYO18B*) further promotes concatenation (Jiu et al., 2019), likely by acting as a structural scaffold that stabilizes the alignment and promotes fusion of neighboring bipolar filaments (Fig.2c). Another protein involved in stabilization of assembly monomers is the chaperone UNC45a (Lehtimaki et al., 2017) (Fig.2c). In addition, coiled-coil segments from folded myosin monomers may engage exposed tail regions within existing filaments via electrostatic interactions, supporting staggered incorporation of new monomers and explaining the characteristic axial staggering observed in thicker filaments (Ricketson et al., 2010) (Fig.2 d).

NM2 filament stability is also controlled by phosphorylations in the coiled-coil and non-helical tail domains of NM2. For example, phosphorylation of NM2-A in Ser1943 (non-helical tail domain) by CK-II decreases the stability of NM2-A filaments (Clark et al., 2008b; Dulyaninova et al., 2007), influencing anteroposterior polarization (Raab et al., 2012). Other amino acids along the assembly domain of NM2-A, 2-B, and 2-C can be phosphorylated by TRPM6/7, also increasing filament instability (Clark et al., 2008a). Regarding NM2-B, earlier work from the Ravid lab showed that PKCζ phosphorylates NM2-B in Ser1937 (non-helical tail domain), controlling filament stability (Even-Faitelson and Ravid, 2006). The amino acid identified in the Ravid study belonged to a poly-Ser cluster, and phosphorylation of Ser1935 at the beginning of the cluster decreases filament stability and impairs anteroposterior polarization in migrating cells (Juanes-Garcia et al., 2015). Interestingly, mutagenic insertion of the poly-Ser cluster in the equivalent position of NM2-A endows NM2-A with NM2-B-like properties in terms of filament stability and anteroposterior polarization competence, highlighting the crucial role of this cluster in the stability of NM2-B filaments compared to NM2-A (Juanes-Garcia et al., 2015). Finally, phosphorylation of amino acids in other regions of the heavy chain of NM2-A may also impart context-specific signals to control NM2-A motor function and/or filament stability (Almeida et al., 2015).

Although the mechanism by which folded NM2 monomers achieve assembly competence is relatively well understood, the processes governing NM2 filament disassembly and the recycling of monomers back into the reservoir pool remain less characterized. A possible hypothesis is that, once detached from F-actin but still in an extended state, the NM2 regulatory light chain (RLC) undergoes dephosphorylation. This conformational change would destabilize the monomer’s association with the filament, allowing it to rapidly refold into its inactive conformation as it is jettisoned out of the filament. The model assumes that this transition occurs during the ATP-bound, actin-unbound phase of the cross-bridge cycle. Additional factors, such as post-translational modifications or protein–protein interactions near the assembly competence domain (Juanes-Garcia et al., 2015; Li et al., 2003), may significantly modulate filament disassembly. Furthermore, mutations that alter interactions among heavy chain subdomains have been reported to affect this pathway (Casas-Mao et al., 2024; Pal et al., 2020; Ricketson et al., 2010). Evidence from FRAP and photo-conversion experiments (Llorente-Gonzalez et al., 2026; Vicente-Manzanares et al., 2007) indicates that NMII monomers released from filaments can either initiate de novo filament formation or laterally integrate into pre-existing structures. This is also a poorly characterized process that could take place in a similar manner to that of initial bipolar filament extension, that is, by adding folded monomers interacting through the tail domain that are further extended by MLCK-dependent phosphorylation of RLC in Ser19. These findings and hypotheses strongly suggest that disassembly and lateral exchange involve conformational folding of the NMII hexamer as it re-enters the reservoir pool, with reincorporation into filaments requiring subsequent phosphorylation and extension.

NM2 bipolar filaments interact with actin microfilaments to build contractile structures that resemble the filament arrays found in striated muscle, but they do so at a much smaller physical and mechanical scale. The force generated by a single NM2 motor is typically in the pN range (Bleicher et al., 2025; Kovacs et al., 2007), similar to the forces estimated for an individual motor in the context of the sarcomere, but the way these forces are organized and summed in each system is fundamentally different. In skeletal muscle, sarcomeres are arranged in large numbers and in defined geometries that promote force additivity. Sarcomeres aligned in parallel across the cross-section of a fiber each generate active stress that sums over the fiber area, yielding macroscopic forces in the millinN–N range (Shimamoto et al., 2009). Sarcomeres connected in series along myofibrils all bear the same force, but their individual length changes accumulate, allowing large overall shortening at essentially constant force without significant loss along the chain (Hinks et al., 2023). By contrast, a typical NM2 mini filament contains on the order of a few tens of NM2 motors, with the specific number depending on the paralog and cellular context (Billington et al., 2013). Outside of organized bundles such as stress fibers (see next section), their activity is distributed in a patchier, network-like fashion rather than in large, strictly parallel arrays. As a result, NMII filaments usually generate localized forces that are a few tens of pN in magnitude, which are well suited to driving local remodeling of the cytoskeleton, adhesions, and extracellular matrix.

Importantly, NM2 interactions with MM2 enable sarcomeric formation. In a series of elegant studies, the Sanger group showed that NM2-A, NM2-B, and NM2-C localize to pre-myofibrils in immature muscle precursors (Wang et al., 2018) together with muscle-specific α-actinin (White et al., 2018). As cells fuse and mature into myotubes, NM2 paralogs disappear from myofibrils and are replaced by MM2 (Wang et al., 2018; White et al., 2014; White et al., 2018). Furthermore, a study from the Burnette group using iPSC-derived cardiomyocytes showed that arc-like stress fibers containing formins and NM2-A and NM2-B act as sarcomere precursors, and the genetic deletion of NM2 paralogs or FHOD3 formin impairs sarcomere and A-band formation, underscoring the crucial role of NM2 in sarcomere development in muscle cells (Fenix et al., 2018). NM2-B also plays a role in cardiac disease, since inherited arrhythmogenic cardiomyopathy caused by mutations in plakophilin 2 can be countered by expression and/or pharmacological activation of NM2-B. Mutant PKP2 induces aberrant actomyosin organization in mouse cardiomyocytes that is corrected by expression and/or activation of NM2-B (Garcia-Quintans et al., 2023), suggesting an important role for NM2 in the maintenance of normal actomyosin organization even in mature muscle cells.

In summary, force generation by MM2 and NM2/SM2 has many similarities, including the existence of an ATP-saving reservoir form and the activating effect of RLC phosphorylation by tissue-specific MLCK isoforms. However, key specific distinctions attune each type of motor to their metazoan function: MM2 forms stable thick filaments that contain MM2 molecules in active, disordered, activatable, and super relaxed (ATP-saving) states; they do not appear in a soluble cytoplasmic form, and forces generated by MM2 arrays are highly additive due to their stability and geometric arrangement with respect to each other, enabling the production of large-scale forces. Conversely, SM2 and specially NM2 form smaller arrays that produce pN-scale forces and form and disassemble filaments dynamically, with MLCK playing an essential role in the formation of the filaments through disruption of the folded, ATP-saving 10S conformation.

## Types of SM2/NM2 structures in smooth muscle and non-muscle cells

MM2 assembles exclusively into sarcomeres in striated muscle cells, displaying a characteristic striated pattern (Burbaum et al., 2021). Some non-muscle myosin II (NM2) isoforms act as early scaffolds for sarcomere assembly in myogenic precursors, and are then downregulated upon differentiation, when MM2 paralogs become the dominant motors supporting the formation of mature sarcomeres (Wang et al., 2018; White et al., 2018).

**Fig. 3:**
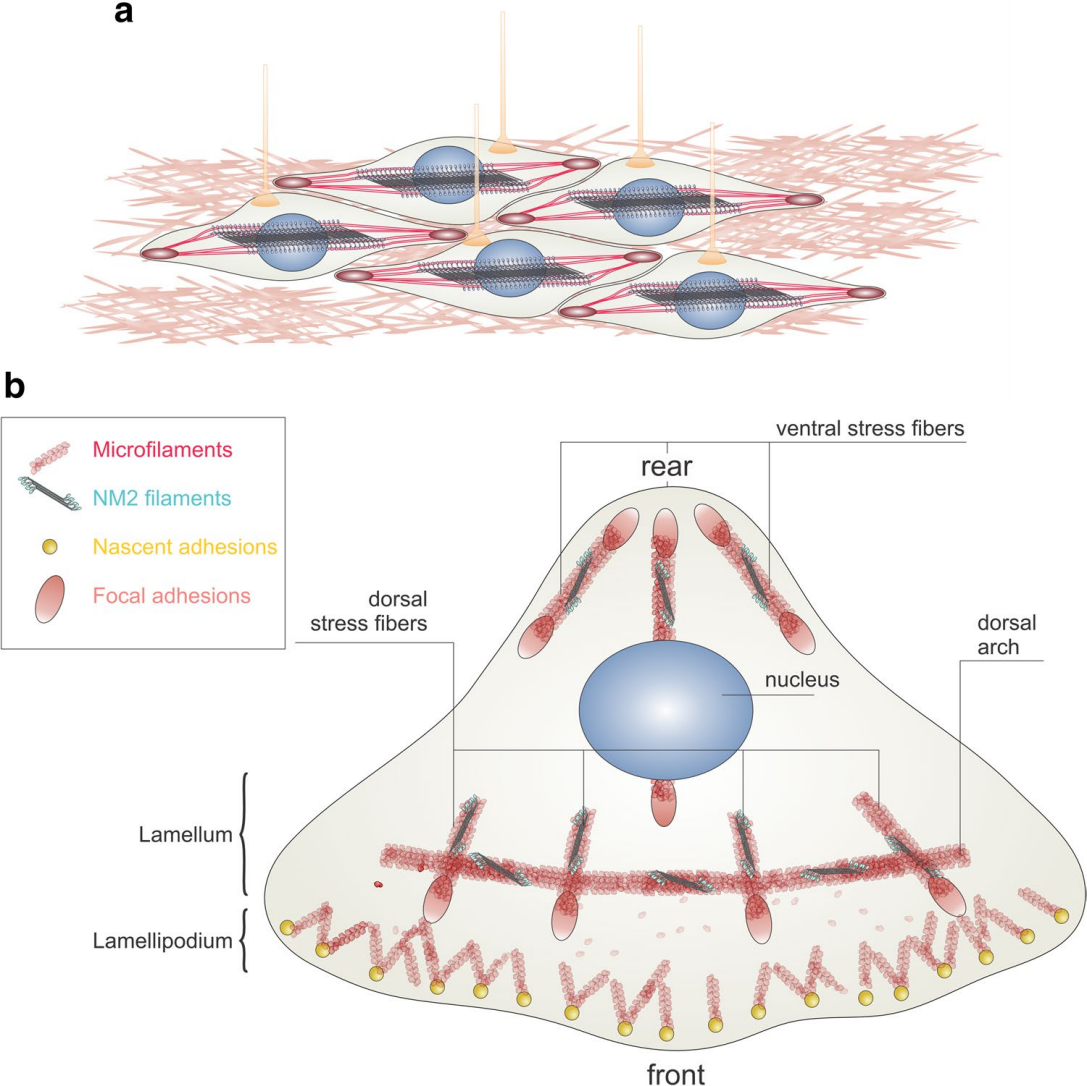
SM2 and NM2 structures in smooth muscle and non-muscle cells, respectively. **a** Smooth muscle cells display polar arrays of SM2 connected to the plasma membrane by F-actin filaments interacting with diffuse membrane plaques. Upon nervous stimulation (yellow growth cones), SM2 undergoes coordinated contraction in a Ca2.^+^-dependent manner. **b **In polarized non-muscle cells, NM2 arrays appear associated with dorsal stress fibers, dorsal arches, and ventral stress fibers. The absence of NM2-A impairs the occurrence of all these structures, whereas NM2-B deletion impairs only the polarity defined by ventral stress fibers. The distribution of NM2 paralogs is not even and has been extensively reviewed elsewhere (Garrido-Casado et al., 2021; Vicente-Manzanares et al., 2009).

In smooth muscle cells, SM2 is the predominant paralog. It assembles into bipolar sheets (Figs. 1b and 3a) attached to the plasma membrane by dense structural junctions. Smooth muscle cell innervation and dense junctions provide coordination for large-scale movement of smooth muscle tissues.

In non-muscle cells, NM2 organization is highly context dependent, varying with both cell type and paralog. In migrating cells, NM2 first appears as small assemblies near the leading edge (in the lamellum, Fig. 3b) that seed higher order actomyosin structures such as stress fibers (Vicente-Manzanares et al., 2011). These nascent structures are enriched in NM2-A, an isoform with rapid turnover and a short duty ratio, defined as the fraction of the chemo-mechanical cycle during which the myosin head remains bound to actin. More central and rear regions of the cell are instead dominated by NM2 paralogs with longer duty ratios, which assemble into more stable bundles specialized for sustained tension (Vicente-Manzanares et al., 2011), including structures involved in nuclear positioning (Thomas et al., 2015).

Analogous NM2-based assemblies have been described in essentially all migrating cell types examined, including neuronal growth cones (Cheng et al., 1992), fibroblasts (Lo et al., 2004), epithelial cells during wound closure (Tamada et al., 2007), cancer cells (Beach et al., 2011), and rapidly moving leukocytes (Jacobelli et al., 2009; Jacobelli et al., 2004; Jacobelli et al., 2010). Experimental evidence indicates that these structures promote focal adhesion stabilization on two-dimensional substrates (Choi et al., 2008), although the kinetics and mechanisms of adhesion growth remain strongly dependent on cell type and on the surrounding microenvironment.

In static epithelial monolayers, NM2 concentrates at the apical–lateral border, where it forms a contractile actin belt that is mechanically coupled to cadherin-based junctions and helps maintain barrier function and coordinated tension across the sheet (Smutny et al., 2010), including during junctional remodeling and YAP-regulated morphogenetic events (Zhang et al., 2020). At the basal surface, cells assemble NM2-containing actin bundles that attach to integrin-containing adhesions, especially on rigid planar substrates (Choi et al., 2008). These structures, commonly referred to as stress fibers, provide a flexible, contractile interface that mediates force transmission to the extracellular matrix.

Stress fibers are typically described as elongated actin–myosin bundles that contain periodic NM2 and actin crosslinker-rich segments and that connect to the extracellular matrix through focal adhesions, enabling them to generate and transmit tension along the ventral surface of the cell (Hotulainen and Lappalainen, 2006; Tojkander et al., 2015; Tojkander et al., 2012). In mesenchymal cells such as fibroblasts or many adherent carcinoma and sarcoma cell lines, these bundles are especially prominent and diversify into related architectures (Fig.3b), including adhesions-linked dorsal fibers, curved transverse arcs that move toward the cell center, and ventral fibers that connect two focal adhesions (Tojkander et al., 2012).

These different stress fiber classes share a core actomyosin machinery but show subtle differences in protein composition, degree of coupling to adhesions, and contribution to traction forces and cell shape control (Tojkander et al., 2012). Live-cell imaging has demonstrated that stress fibers can arise from the progressive reorganization of smaller NM2-associated actin bundles formed near the leading edge (Jiu et al., 2019), with dorsal fibers and arcs acting as precursors that remodel into ventral fibers as they connect to and mature focal adhesions (Choi et al., 2008).

By linking integrin-based adhesion sites to extended actin bundles and, through perinuclear actin cables and nucleo-cytoskeletal linkers, to the nuclear envelope, stress fibers form part of a continuous mechanical pathway that conveys external forces and matrix stiffness cues to the interior of the cell (González-Martín et al., 2025). This physical coupling underpins a central tenet in mechanobiology: tension generated and transmitted by NM2 at junctions and adhesions feeds into signaling pathways and nuclear mechanics that adjust gene expression programs, including those that control YAP/TAZ activity (Piccolo et al., 2023) and other mechanoresponsive transcription factors, e.g., MRTF (Speight et al., 2016), Nkx2.5 (Dingal et al., 2015) or NF-κB (Liu et al., 2007).

**Fig. 4:**
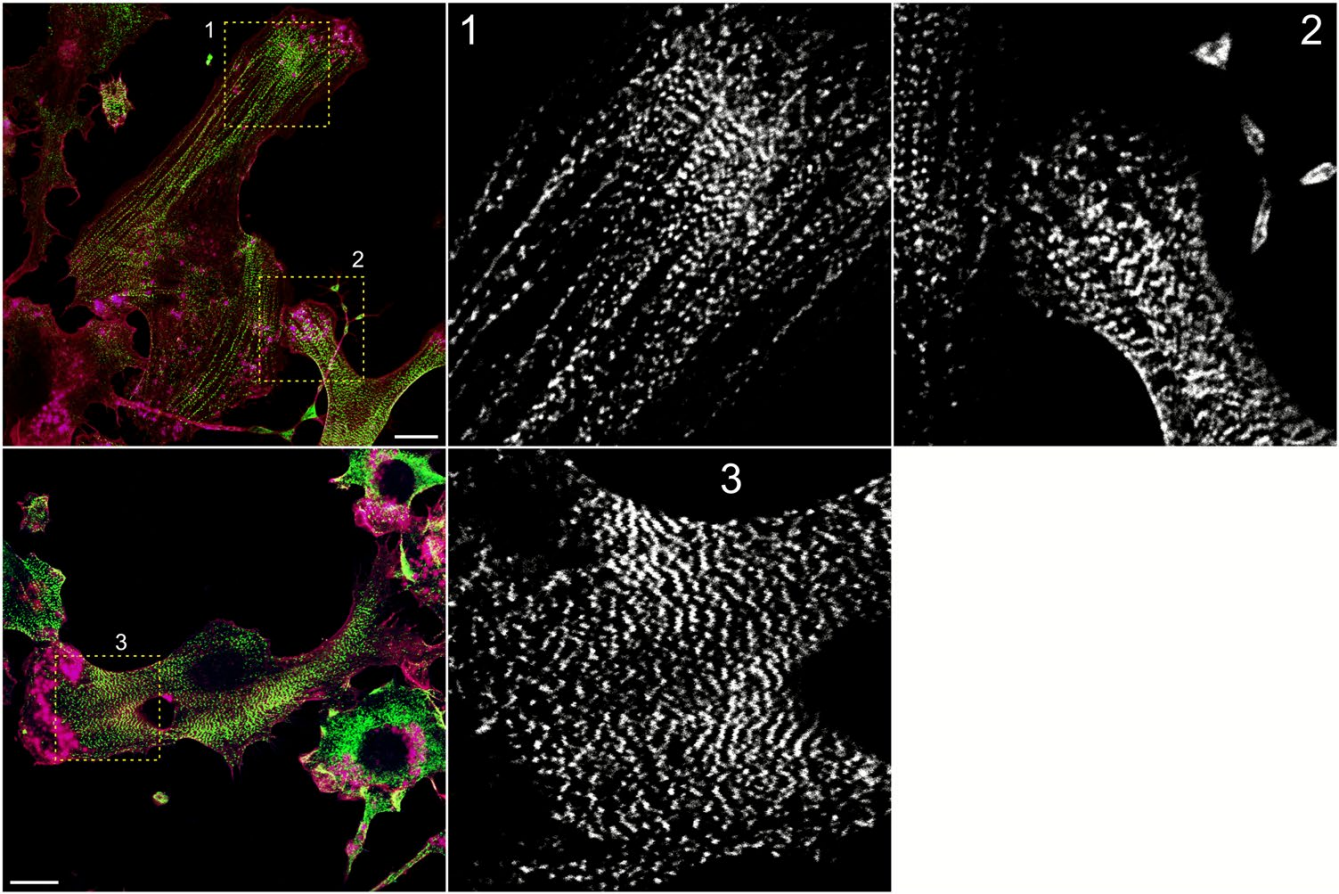
Sarcomere-like features of NM2-A in hematopoietic cells. Human monocyte-derived dendritic cells cultured for 7 days in the presence of IL-4 (10 ng/mL) and GM-CSF (20 ng/mL) were allowed to adhere to 20 µg/mL fibronectin for 30 min, fixed, and stained for NM2-A (green), F-actin (red), and focal adhesion protein vinculin (blue). Magenta represents the co-localization of F-actin and vinculin; scale bars = 10 µm. Yellow boxes are magnified on black and white panels (NM2-A only). Note the sarcomere-like arrangement of NM2-A stacks

In cell types that do not build prominent stress fibers, NM2 still forms distinct supramolecular assemblies, but their geometry and linkage to adhesion sites differ from the classic ventral bundles seen in fibroblasts. For example, macrophages and dendritic cells can organize NM2 into short chains or clustered arrays of filaments that display a partially bipolar pattern reminiscent of sarcomeric segments (Fig. 4). These structures typically reside within the cortical network and show only indirect or transient connections to substrate-anchored adhesion complexes.

Other cells, e.g., primary lymphocytes and many leukemic lines, lack both extended stress fibers and NM2 filament stacks. In these cells, NM2 tends to appear as small, dispersed filaments or filament fragments embedded in the actin-rich cortex, where they likely engage nanoscale adhesion assemblies or receptor clusters that fall below the resolution of conventional microscopy.

Subcortical non-muscle myosin II (NM2) constitutes a fundamental element of the actomyosin cortex, a narrow (~ 60 to 150 nm) layer composed of actin filaments and associated binding proteins located beneath the inner leaflet of the plasma membrane. This cortical structure is central to the generation and regulation of mechanical tension during diverse physiological processes. For instance, cortical tension increases during mitosis and diminishes during interphase. The thickness of the cortical actin layer is primarily governed by actin polymerization regulators such as CAPZ, DIAPH1, and cofilin (Chugh et al., 2017). Perturbation of these factors alters cortical actin thickness—either increasing or decreasing it—yet consistently results in reduced cortical tension. This phenomenon reflects the dependence of tension generation on filament length: intermediate-length actin filaments are optimally suited to support tension buildup, irrespective of NM2 enrichment at these sites (Chugh et al., 2017). The mechanistic basis lies in the favorable stoichiometric ratio between NM2 molecules and actin binding sites, which facilitates bundle compaction against the plasma membrane. Consequently, the degree of connectivity among actin filaments dictates contraction dynamics, producing either filament shortening and expansion reminiscent of sarcomeric organization or filament buckling (Ennomani et al., 2016).

Advanced live-cell imaging has revealed that, in interphase, subcortical actin and NM2 assemble into distinct yet intertwined ring structures that merge upon entry into mitosis (Truong Quang et al., 2021). Within the NM2 ring, filaments are oriented orthogonally relative to the plasma membrane, with one pole directed toward the membrane and the opposite pole facing the nucleus. During mitosis, overlap between the NM2 and actin rings increases in a manner dependent on NM2 activation. These observations support a model in which, during interphase, cortical NM2 filaments interact with actin predominantly through the membrane-proximal pole, while the distal pole remains disengaged in the cytoplasm. Upon mitotic activation, both poles become engaged, thereby enhancing actomyosin contractile output in this region. Complementary biochemical and cell-based studies further indicate that specific regions of the NM2 rod domain can bind anionic lipids, leading to localized enrichment at the plasma membrane (Liu et al., 2016). Such lipid-mediated recruitment may provide an additional mechanism for spatially modulating NM2–actin interactions, potentially in response to extracellular cues.

This could be important in the context of amoeboid cell migration, which is a low-adhesion mode of cell movement in which cells advance by continuously remodeling their shape and relying heavily on cortical actomyosin contractility rather than on strong, stable adhesions to the extracellular matrix. This behavior is particularly prominent in leukocytes, amoebae, and many invasive tumor cells, which can squeeze through complex 3D environments with minimal matrix degradation (Friedl and Wolf, 2010). A prominent variant is bleb-based, or blebby, migration (Charras et al., 2008), where propulsion is dominated by cycles of membrane bleb formation and retraction: local weakening or detachment of the actin cortex allows intracellular pressure generated by myosin II contraction to push the plasma membrane outward, after which actin repolymerization and cortex reassembly stabilize and retract the bleb, effectively pulling the cell body forward (Charras et al., 2005; Kelkar et al., 2020). Bleb-driven motility is favored in confined, low-adhesion settings and can interconvert with more actin protrusion–dominated modes (Liu et al., 2015), allowing cells to rapidly adapt their migration strategy to the mechanical and adhesive properties of their surroundings. In this context, fast regulation of cortical NM2 in response to chemo-mechanical cues from low-adhesivity microenvironments is likely to play a crucial role in the rapid readaptation of highly motile cells to their surroundings.

## Myosin pathologies and mechanotherapy

### Cardiac MM2 pathologies and a brief overview of novel treatments

Mutations in genes encoding myosin II heavy chains are strongly associated with human disease. The most prominent example involves mutations in the *MYH7 *gene, which encodes β-cardiac myosin (βCM2). Loss-of-function variants reduce ATPase activity and impair cross-bridge cycling, ultimately triggering dilated cardiomyopathy, or DCM (Colegrave and Peckham, 2014). At the systemic level, DCM manifests as ventricular dilation, systolic dysfunction, progressive heart failure, and overall weakening of the myocardium (Myers et al., 2024; Wieczorek, 2024). Conversely, gain-of-function mutations increase ATPase activity and promote sarcomeric hypercontractility (Marian, 2021), leading to hypertrophic cardiomyopathy, or HCM. Macroscopic features of HCM include thickening of the ventricular walls and interventricular septum, diastolic dysfunction, mitral regurgitation, arrhythmia, and an elevated risk of sudden cardiac death (Maron et al., 2022).

**Fig. 5:**
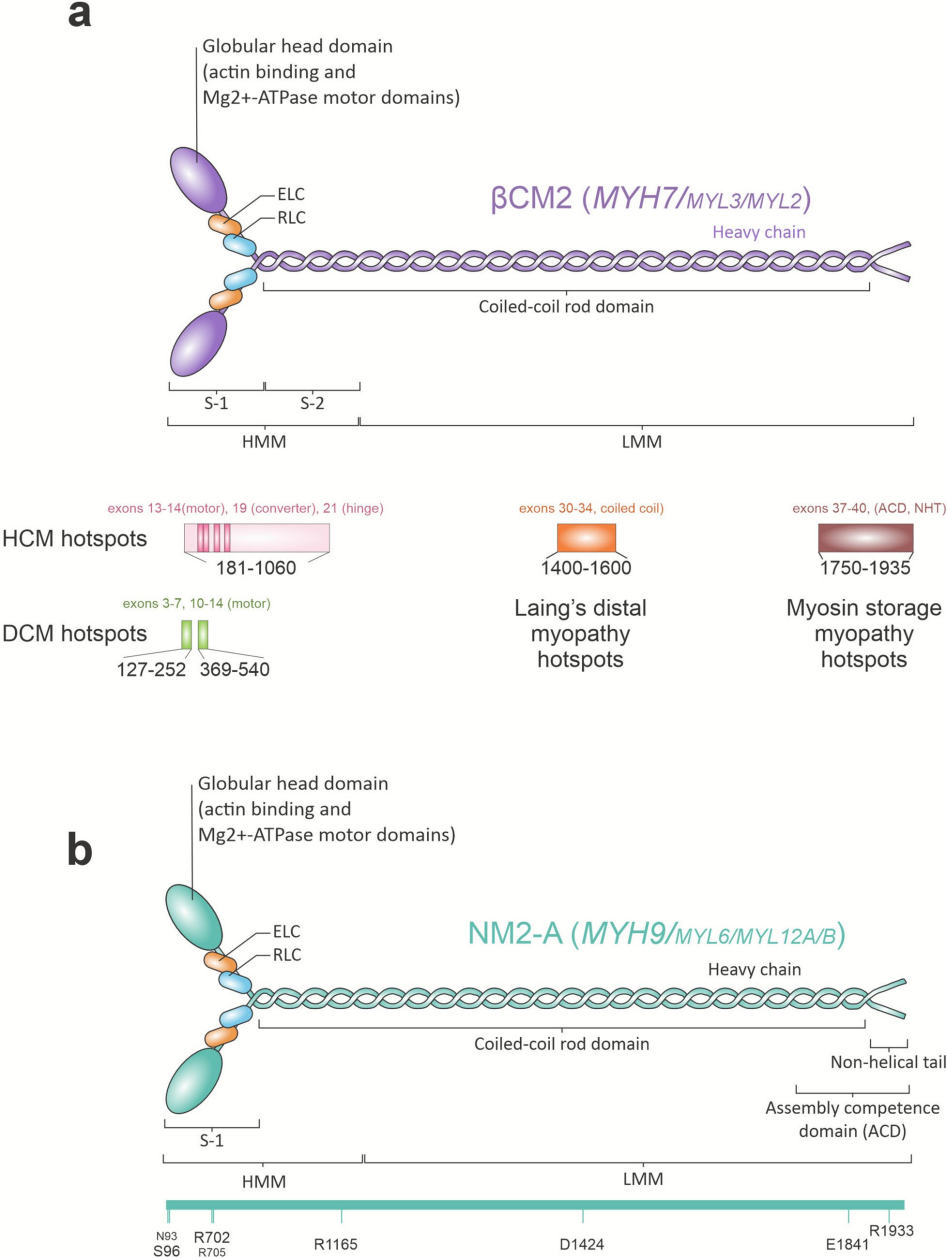
Mutational hotspots in *MYH7* and *MYH9* genes. **a** *MYH7* hotspots for hypertrophic cardiomyopathy (HCM) are shown in pink, dilated cardiomyopathy (DCM) in green, and distal myopathies in orange and brown. Regions with higher probability are shown in pale colors, and clustered hotspots are shown in intense colors. **b** Representative mutations causing *MYH9*-RD in NM2-A. See ref. (Asensio-Juárez et al., 2020) for additional details

Documented missense mutations in *MYH7 *associated with DCM include S532P, R870H, E924K, R1045C, R1434H, and many others (Jansen et al., 2024). DCM is also triggered by the introduction of nonsense mutations that truncate the coiled-coil domain (de Frutos et al., 2022). There are two major hotspots between amino acids 127–252 (encoding approximately exons 3–7) and 369–550 (exons 10–14, Fig.5a), with the rest of the mutations scattered throughout different positions of the protein (de Frutos et al., 2022; Jansen et al., 2024). In general, these mutations are expected to reduce thick filament integrity or diminish contractile output (Colegrave and Peckham, 2014; Pires et al., 2025).

*MYH7* is, together with *MYBPC3*, the most frequently mutated gene in HCM, but no single mutation is particularly abundant (Pires et al., 2025). The main HCM-related mutation hotspot lies between amino acids 181–937 (Garcia Hernandez et al., 2022; Pires et al., 2025), although some authors localize it between amino acids 370–1060 (Vepsäläinen et al., 2022). Most mutations in this hotspot affect amino acids encoded in exons 13, 14, corresponding to the motor domain; exon 19, encoding part of the converter domain; and exon 21, encoding the hinge, ELC binding site. Figure 5a displays the outer margins of the consensus region. Examples include mutations in R403(Q/W). These are very severe and are often used to model the disease (Ghanta et al., 2025; Steczina et al., 2025). Overall, HCM-triggering variants increase the ATPase activity of myosin and elevate the fraction of active heads within the sarcomere, thereby reducing the proportion of heads in the super-relaxed (SRX) state.

Another subset of *MYH7 *mutations underlies rarer conditions, such as left ventricular non-compaction, Laing distal myopathy, and myosin storage myopathy (Gao et al., 2024). Left ventricular non-compaction (LVNC) is a rare condition caused by mutations to *MYH7*, mostly loss of function, but no mutation hotspot has been identified to date.

Laing distal myopathy primarily impairs mobility of distal muscles, including those of the toes and calves (Lamont et al., 2014). It mainly affects amino acids in the coiled-coil domain (Fig.5a). Interestingly, Laing’s myopathy and DCM may emerge from mutations in the same amino acid, e.g., R1500 within the coiled coil domain. This amino acid can be mutated to Pro (R1500P) or Trp (R1500W). Both mutations make βCM2 more unstable in thermodynamic terms, but filaments made of R1500P are more unstable than those containing R1500W (Armel and Leinwand, 2010). In contrast, myosin storage myopathy is characterized by progressive muscle weakness and is triggered by mutations within exons encoding the coiled-coil domain of βCM2 (Fig.5a), notably exons 38 and 39 (Bahout et al., 2025). Reported examples include R1845W, E1883K, E1886K, E1902K, and N1918K. These substitutions alter electrostatic interactions, destabilizing the thick filament core and promoting the formation of misfolded βCM2 aggregates (Tajsharghi and Oldfors, 2013). Table 2 displays some examples of mutations found in patients of those diseases.

**Table 2:** Examples of *MYH7* mutations found in allele carriers of the indicated diseases

Disease	Representative mutations	Gain–loss of function	Main features
	R403Q	Gain	Severe HCM
**HCM**	R403W	Gain	Aggressive, early onset
	R453C	Gain	Reported in familial HCM
	V606M	Gain	Variable severity
	R719W	Gain	Variable penetrance
	R723G	Gain	High risk of sudden death
	I736T	Gain	ELC binding
	G741R	Gain	ELC binding
	R787H	Gain	ELC binding
	S532P	Loss	Structural instability
**DCM**	R870H	Loss	Structural instability
	E924K	Loss	Low ATPase activity
	R1045C	Loss	Early onset
	R1434H	Loss	Filament instability
	R243H	Loss	Impaired actin binding
**LVNC**	N315R	Loss	Low ATPase
	R870H	Loss	Filament instability
	R1359C	Loss	Filament instability
	R1712W	Loss	Filament instability
	R1424H	Loss	Filament instability
**Laing’s distal myopathy**	R1463W	Loss	Filament instability
	R1500P	Loss	Filament instability
	E1508K	Loss	Filament instability
	E1560P	Loss	Filament instability
	L1793P	Loss	Filament instability
**Myosin storage myopathy**	R1845W	Loss	Filament instability
	E1883K	Loss	Filament instability
	R1893H	Loss	Filament instability
	H1901K	Loss	Filament instability
	N1918K	Loss	Filament instability

Also indicated are whether the mutation produces gain- or loss-of-function and the molecular effect of the mutation on βCM2 function and/or filament status

Mutations in other sarcomeric components involved in force generation may also contribute to dilated or hypertrophic cardiomyopathy. In DCM, mutations in various troponin isoforms can reduce calcium sensitivity and disrupt thick–thin filament interactions (Ho et al., 2009; Ohte et al., 2009; Parvari and Levitas, 2012). Similarly, mutations in*TPM1*can impair actin–myosin interactions, promoting ventricular dilation (Parvari and Levitas, 2012; Redwood and Robinson, 2013). In contrast, mutations in these same components, as well as in actin or myosin-binding protein C, have been implicated in HCM (Redwood and Robinson, 2013; Schuldt et al., 2021; Tudurachi et al., 2023).

In summary, DCM arises from reduced contractile force output due to loss-of-function mutations in MYH7 or other sarcomeric proteins critical for force generation. Conversely, HCM results from exaggerated force output driven by gain-of-function mutations in *MYH7* or related sarcomeric proteins.

Currently, no treatments directly target the underlying mutations responsible for dilated cardiomyopathy (DCM). Management instead focuses on symptomatic control, for example with ACE inhibitors to reduce afterload or beta-blockers to improve ventricular function. In selected cases, medical devices such as implanted cardioverter-defibrillators or cardiac resynchronization therapy may be beneficial. In advanced stages of the disease, heart transplantation often becomes necessary (Sorella et al., 2025). New, advanced therapies will emerge in response to a better understanding of the correlation between genotype (*MYH7 *mutations) and phenotype (DCM), which is still underdeveloped. Novel mechanisms to promote myocardial regeneration may include the use of exosomes and/or microRNA to reduce fibrotic damage (Sinagra et al., 2019). Finally, advances in DNA editing may make it feasible to promote myocardial regeneration using edited stem cells, although such approaches are still in their infancy and face similar challenges to other regenerative schemes to treat cardiac failure and/or stroke.

By contrast, hypertrophic cardiomyopathy (HCM) has a specific pharmacological therapy. The recently developed βCM2 inhibitor, 2-[2-(4-(pyridin-2-yl)phenoxy)acetamido] benzoic acid, termed mavacamten (Green et al., 2016), stabilizes βCM2 in the super-relaxed (SRX) state (Anderson et al., 2018; Rohde et al., 2018), thereby reducing hypercontractility. Precise dosing is essential, as excessive inhibition may compromise cardiac output (Scholtz et al., 2025). Mavacamten is highly specific for cardiac myosin and received FDA approval in April 2022 (EMA approval in June 2023) following extensive clinical evaluation (Liao et al., 2024). It has been demonstrated to be safe and effective in patients with class II–III symptomatic obstructive HCM, representing the first targeted therapy for a disease caused by mutations in motor proteins of the myosin superfamily. Whether mavacamten may benefit patients with genetic alterations underlying Laing distal myopathy or myosin storage myopathy remains to be determined.

### MYH9-RD and other NM2 pathologies

Mutations in non-muscle myosin II (NM2) also cause disease. These conditions, collectively termed *MYH9*-related diseases (*MYH9*-RD), are rare autosomal dominant genetic disorders, that is, cells express one mutant allele and one wild-type allele. They consistently present with macrothrombocytopenia, characterized by large, scarce platelets with impaired functionality (Heath et al., 2001; Kelley et al., 2000; Lalwani et al., 2000; Seri et al., 2000; Seri et al., 2003). In addition, some patients develop extra-hematological manifestations, most notably mid-life renal failure, congenital deafness, and early-onset cataracts (Althaus and Greinacher, 2009). Diagnosis typically involves identification of macrothrombocytopenia, followed by detection of Döhle-like inclusion bodies in neutrophils and confirmation by whole-genome sequencing (WGS)(Althaus and Greinacher, 2010).

Most *MYH9*-RD mutations (reviewed in (Asensio-Juárez et al., 2020)) either do not alter the enzymatic function of NM2-A (Casas-Mao et al., 2024; Llorente-Gonzalez et al., 2026) or represent loss-of-function variants (Kim et al., 2005) (Fig.5b). A general clinical consensus suggests that mutations in exons encoding the head domain produce more severe phenotypes than those affecting the coiled-coil domain (Pecci et al., 2014), a pattern reminiscent of *MYH7 *mutations in muscle and cardiac tissue. A recent study from our group showed that, although mutations in both regions of the molecule induce protein aggregation, the properties of these aggregates differ markedly. Head-domain mutations, though rare, generate large amorphous aggregates in both adherent and hematopoietic cells. These aggregates impair the function of the wild-type protein, other NM2 paralogs, and associated scaffolding proteins such as the folding chaperone UNC45a. In contrast, mutations in the coiled-coil or tail domain aggregate exclusively in hematopoietic cells. These aggregates are structurally distinct from those caused by head-domain mutations and do not interfere with wild-type NM2-A, other paralogs, or chaperones (Llorente-Gonzalez et al., 2026).

Currently, there is no specific therapy that directly targets the underlying mutations in *MYH9*-related disease (*MYH9*-RD). Management is therefore supportive and symptom-oriented. Hematological care includes regular monitoring of platelet counts, administration of tranexamic acid, and platelet transfusions in cases of severe bleeding. Renal involvement is typically managed with ACE inhibitors, although advanced disease may necessitate kidney transplantation. Hearing loss can be addressed with hearing aids or cochlear implants, while cataracts are treated surgically (Althaus and Greinacher, 2010). However, the lack of basic knowledge on the causal factors of extra-hematological manifestations limits the design of novel treatments.

### Role of NM2 in cancer and the biology of substance abuse

Non-muscle myosin II (NM2) is a central regulator of cellular mechanics, with its capacity to generate contractile force conferring a pivotal role in dynamic biological processes and disease states. In cancer, for instance, tumors exhibit mechanical properties distinct from their surrounding microenvironment, facilitating dissemination and metastatic colonization. Similarly, in autoimmune disease, aberrant cell migration enables immune cells to infiltrate inappropriate tissues and inflict self-damage. Importantly, the activation state of NM2 serves as a functional readout of tissue mechanics, with increased rigidity correlating with tumor aggressiveness, as exemplified in glioblastoma (Miroshnikova et al., 2016).

Although mutations or copy number alterations in NM2 monomers are not direct oncogenic drivers, NM2 regulation represents a point of convergence for multiple signaling pathways frequently altered in cancer. This makes NM2 an attractive candidate for adjuvant therapeutic intervention (Gandalovicova et al., 2017). Blebbistatin is a well-characterized NM2 inhibitor widely used to block actomyosin contraction in vitro (Straight et al., 2003). Despite its apparent tolerability in selected contexts including the treatment of rheumatoid arthritis (Lee et al., 2023), ulcerative colitis (Zhao et al., 2015), and substance abuse (Young et al., 2016; Young et al., 2017), blebbistatin is likely unsuitable for clinical use due to its reported cardiotoxicity (Brack et al., 2013). Encouragingly, next-generation blebbistatin derivatives with negligible cardiac side effects have demonstrated efficacy in pre-clinical glioblastoma models (Kenchappa et al., 2025). Unexpectedly, their therapeutic benefit appears to derive not from inhibition of cell migration but from modulation of mitochondrial dynamics. Specifically, NM2 depletion (Kage et al., 2022) or pharmacological inhibition (Kenchappa et al., 2025) disrupts mitochondrial fission, leading to the accumulation of reactive oxygen species and induction of ferroptosis (Kenchappa et al., 2025). Moreover, NM2 inhibition enhances oncogene dependency in cancer cells and synergizes with targeted therapies, such as PDGFR inhibitors (Kenchappa et al., 2025). Nevertheless, whether these findings can be extended to other forms of cancer remains uncertain, particularly, given the reports that NM2-A is a tumor suppressor in squamous carcinoma (Conti et al., 2015; Schramek et al., 2014), glioblastoma (Picariello et al., 2019), and melanoma (Singh et al., 2020). In addition, there is evidence that reducing NM2-A may increase the levels of NM2-B and/or 2-C (Schramek et al., 2014), which may have unpredictable effects on the mechanical and/or biochemical behavior of the cells.

Beyond cancer, NM2 also exerts critical control over actin cytoskeletal remodeling in the nervous system. NM2-B has been implicated in dendritic spine remodeling during hippocampal spinogenesis (Hodges et al., 2011), cortical long-term potentiation (Rex et al., 2010), and in response to methamphetamine exposure in basolateral amygdala neurons (Young et al., 2020). Notably, a novel blebbistatin analog, developed alongside the glioblastoma-targeting compound (Kenchappa et al., 2025), attenuates actin dynamics in response to methamphetamine and reduces drug-seeking behavior in animal models, without affecting responses to cocaine or fear-induced conditioning (Radnai et al., 2025). These findings, coupled with the apparent lack of toxicity of these analogues, highlight the potential of NM2-targeted therapies to revolutionize treatment strategies for addiction.

## Concluding remarks

From its first description as the “thick substance” in muscles, myosin has evolved from a biochemical curiosity into a central player revealed by molecular, electrophysiological, and imaging breakthroughs. Today, the ability to target myosin-2 in paralog- and in tissue-specific ways is reshaping treatments for conditions as diverse as heart malformations, cancer, and addiction. Molecular motors have always powered the inner life of cells, but these advances put myosin-2 back in the spotlight—heralding what may well be a golden era of discovery with rapid impact on patient care.

## Data Availability

The original microscopy images shown in Fig. 4 will be made available by the authors upon reasonable request.
